# Accuracy of the Sequential Organ Failure Assessment Score for In-Hospital Mortality by Race and Relevance to Crisis Standards of Care

**DOI:** 10.1001/jamanetworkopen.2021.13891

**Published:** 2021-06-18

**Authors:** William Dwight Miller, Xuan Han, Monica E. Peek, Deepshikha Charan Ashana, William F. Parker

**Affiliations:** 1 Section of Pulmonary and Critical Care Medicine, University of Chicago, Chicago, Illinois; 2 Section of General Internal Medicine, University of Chicago, Chicago, Illinois; 3 MacLean Center for Clinical Medical Ethics, University of Chicago, Chicago, Illinois; 4 Center for the Study of Race, Politics, and Culture, University of Chicago, Chicago, Illinois; 5 Division of Pulmonary, Allergy and Critical Care Medicine, Duke University, Durham, North Carolina

## Abstract

**Importance:**

Crisis Standards of Care (CSC) are guidelines for rationing health care resources during public health emergencies. The CSC adopted by US states ration intensive care unit (ICU) admission using the Sequential Organ Failure Assessment (SOFA) score, which is used to compare expected in-hospital mortality among eligible patients. However, it is unknown if Black and White patients with equivalent SOFA scores have equivalent in-hospital mortality.

**Objective:**

To investigate whether reliance on SOFA is associated with bias against Black patients in CSC.

**Design, Setting, and Participants:**

This cohort study was conducted using data from the eICU Collaborative Research Database of patients admitted to 233 US ICUs in 2014 to 2015. Included individuals were Black and White adult patients in the ICU, who were followed up to hospital discharge. Data were analyzed from May 2020 through April 2021.

**Exposure:**

SOFA scores at ICU admission.

**Main Outcomes and Measures:**

Hierarchical logistic regression with hospital fixed effects was used to measure the interaction between race and SOFA as a factor associated with in-hospital mortality, as well as the odds of death among Black and White patients with equivalent priority for resource allocation according to the SOFA-based ranking rules of 3 statewide CSC (denoted A, B, and C) under shortage conditions that were severe (ie, only patients with the highest priority would be eligible for allocation), intermediate (ie, patients in the highest 2 tiers would be eligible for allocation), or low (ie, only patients with the lowest priority would be at risk of exclusion).

**Results:**

Among 111 885 ICU encounters representing 95 549 patients, there were 16 688 encounters with Black patients (14.9%) and 51 464 (46.0%) encounters with women and the mean (SD) age was 63.3 (16.9) years. The median (interquartile range) SOFA score was not statistically significantly different between Black and White patients (4 [2-6] for both groups; *P* = .19), but mortality was lower among Black individuals compared with White individuals with equivalent SOFA scores (odds ratio [OR], 0.98; 95% CI, 0.97-0.99; *P* < .001). This was associated with lower mortality among Black patients compared with White patients prioritized for resource allocation in 3 CSC under shortage conditions that were severe (system A: OR, 0.65; 95% CI, 0.58-0.74; *P* < .001; system B: OR, 0.70; 95% CI, 0.64-0.78; *P* < .001; system C: OR, 0.73; 95% CI, 0.67-0.80; *P* < .001), intermediate (system A: OR, 0.73; 95% CI, 0.67-0.80; *P* < .001; system B: OR, 0.83; 95% CI, 0.77-0.89; *P* < .001; system C: OR, 0.82; 95% CI, 0.77-0.89; *P* < .001), and low (system A: OR, 0.83; 95% CI, 0.77-0.89; *P* < .001; system C: OR, 0.86; 95% CI, 0.81-0.92; *P* < .001; not applicable for system B, which had fewer tiers). When SOFA-based ranking rules were adjusted for Black patients to simulate equitable allocation based on observed mortality, the proportion upgraded to higher priority ranged from 379 Black patient encounters (2.3%) in low shortage conditions to 2601 Black patient encounters (15.6%) in severe shortage conditions.

**Conclusions and Relevance:**

This study found that SOFA scores were associated with overestimated mortality among Black patients compared with White patients, and this was associated with a structural disadvantage for Black patients in CSC allocation systems. These findings suggest that guidelines should be revised to correct this inequity and alternative methods should be developed for more equitable triage.

## Key Points

**Question:**

Is reliance on the Sequential Organ Failure Assessment (SOFA) score to estimate the risk of in-hospital mortality associated with bias against Black patients in Crisis Standards of Care (CSC)?

**Findings:**

In this cohort study of 95 549 patients in the intensive care unit, in-hospital mortality was lower among Black patients compared with White patients who had equivalent SOFA scores. This overestimation of mortality risk for Black patients was associated with deprioritization of Black patients in CSC.

**Meaning:**

This study's findings that SOFA is associated with overestimated mortality among Black patients compared with White patients suggest that CSC are associated with systematic deprioritization of Black patients.

## Teaser

This cohort study compares mortality among Black and White patients with equivalent Sequential Organ Failure Assessment (SOFA) Scores and their odds of death with equivalent priority in Crisis Standards of Care.

## Introduction

Crisis Standards of Care (CSC) direct ethical decision-making by health systems when public health emergencies overwhelm these systems’ resources. The COVID-19 pandemic had the potential to overwhelm available health care resources, prompting policymakers to develop and revise CSC.^1,2^ All CSC in the United States assert that when intensive care unit (ICU) resources are overwhelmed, first-come, first-serve care should be abandoned and resources should be allocated to patients most likely to survive.^1,2,3,4^ To accomplish this, current CSC compare the likelihood of survival among competing patients using the Sequential Organ Failure Assessment (SOFA) score.^1^ Patients with the lowest SOFA scores are predicted to have the highest likelihood of survival with appropriate care and are therefore given highest priority for resources. However, it is unknown if individuals of different races with equivalent SOFA scores have equivalent probabilities of survival.

Numerous objective prediction models have perpetuated racial bias in consumer lending, law enforcement, and health care.^5,6,7^ This has contributed to concerns that SOFA could be similarly biased. The SOFA score is a composite measure of organ dysfunction based on blood pressure, hypoxemia, creatinine, bilirubin, platelet count, and the Glasgow Coma Scale. It has been validated as a predictor associated with in-hospital survival in diverse patient populations with various illnesses.^8,9,10,11,12,13,14,15,16,17,18^ However, there are known racial variations in the variables used in SOFA. Creatinine, in particular, is known to have a higher mean level among Black patients compared with White patients who have equivalent kidney function.^19,20^ However, whether these observed mean differences translate into higher total SOFA scores and deprioritization of Black patients in triage protocols is unknown. If SOFA overestimates organ dysfunction for Black patients, then it may underestimate their survival compared with that of other patients and unfairly exclude them from receiving scarce resources under CSC.

Therefore, we investigated whether using SOFA is associated with deprioritization of Black patients in currently adopted CSC, which assign higher priority based on lower SOFA. We did this by evaluating survival associated with SOFA at the time of ICU triage for Black and White patients in a large, multicenter, US patient cohort. We hypothesized that equivalent SOFA scores would be associated with higher survival among Black patients compared with White patients, which would be associated with systematic misclassification of Black patients out of the highest priority tiers and into lower priority tiers of CSC.

## Methods

This cohort study was granted exemption from review by the University of Chicago Biological Sciences Division and University of Chicago Medical Center institutional review board given that it used deidentified, publicly available data. All data were certified by Privacert to meet safe harbor standards under the US Health Insurance Portability and Accountability Act, and therefore the University of Chicago Biological Sciences Division and University of Chicago Medical Center institutional review board determined that informed patient consent was not applicable for this study.^21^ It followed the Strengthening the Reporting of Observational Studies in Epidemiology (STROBE) reporting guideline.

### Data Source and Study Population

We performed a retrospective cohort analysis of deidentified patient data from the eICU Collaborative Research Database. This data set has been described previously.^21,22^ Briefly, it includes 200 859 patient encounters for 139 367 unique patients admitted to ICUs in 208 US hospitals from 2014 to 2015. Patients were followed up until hospital discharge. Records for adult patients (ie, those ages ≥18 years) were included if the record represented the first ICU stay during a hospitalization, if at least 1 SOFA variable was recorded within 24 hours of ICU admission, if in-hospital mortality was documented, and if race was recorded in the electronic health record as Black or White. Individuals of other races were excluded owing to low numbers. Ethnicity was not available as a separate field. Records were excluded from hospitals that treated fewer than 10 Black and 10 White patients to allow for characterization of within-hospital variation of mortality between patients of different races and SOFA scores. Records were excluded for patients admitted to the ICU from another hospital or ICU to avoid confounding by missing variables during patient stabilization.

### SOFA Calculation and Tier Definitions

Under CSC, triage personnel calculate SOFA scores and assign priorities to patients who meet usual indications for ICU resources, such as need for mechanical ventilation or vasopressors.^2,23,24^ Therefore, ICU triage is a critical time point to evaluate when quantifying outcomes associated with CSC. To evaluate the association between SOFA and mortality at this time point, we calculated SOFA scores using components available 24 hours before and after ICU admission. When multiple SOFA components were available, we used the value associated with the highest SOFA score. Missing values were imputed as 0 (ie, reference range), a standard practice.^25^ A detailed description of our SOFA calculation is described in eAppendix 1 in the Supplement.

The SOFA score is a continuous variable with 6 components, each scored over ranges from 0 to 4, generating a cumulative total of from 0 to 24. Priority is assigned by CSC based on SOFA-derived tiers (Table 1). Multiple tier systems have been proposed, and the different tier thresholds in these systems may be associated with increased or decreased racial disparities in the raw SOFA scores. To evaluate this, we assessed 3 tier systems that were widely imitated owing to publication in prominent journals and availability early in the COVID-19 pandemic, when institutional and statewide protocols were being developed.^2,3,4^ The first system (ie, system A, developed in Pennsylvania) divides SOFA scores into 4 tiers, with SOFA scores less than 6 assigned highest priority and SOFA scores of 12 or greater given lowest priority. The second system (ie, system B, developed in New York) divides SOFA scores into 3 tiers, with a less restrictive threshold to qualify for highest priority (ie, SOFA score < 8) but an equivalent threshold for lowest priority (ie, SOFA score ≥ 12). The third system (ie, system C, developed in Maryland) divides SOFA scores into 4 tiers, with the least restrictive thresholds for highest priority (ie, SOFA score  < 9) and lowest priority (ie, SOFA score ≥ 15).

**Table 1.** Number of Patient Encounters and Mortality by Priority Tier in Crisis Standards of Care Systems

System	Patient encounters, No. (%)		*P* value	Patient encounters resulting in Mortality, No. (%)		*P* value
	White (n = 95 197)	Black (n = 16 688)		White (n = 8517)	Black (n = 1365)	
A^a^^,^^b^						
Priority 1	64 774 (68.0)	11 163 (66.9)	.03	2438 (3.8)	302 (2.7)	<.001
Priority 2	18 869 (19.8)	3451 (20.7)		2345 (12.4)	376 (10.9)	.01
Priority 3	7820 (8.2)	1405 (8.4)		1864 (23.8)	368 (26.2)	.06
Priority 4	3734 (3.9)	669 (4.0)		1870 (50.1)	319 (47.7)	.27
B^c^						
Priority 1	78 979 (83.0)	13 764 (82.5)	.30	4011 (5.1)	546 (4.0)	<.001
Priority 2	12 484 (13.1)	2255 (13.5)		2636 (21.1)	500 (22.2)	.27
Priority 3	3734 (3.9)	669 (4.0)		1870 (50.1)	319 (47.7)	.27
C^a^^,^^d^						
Priority 1	83 643 (87.9)	14 614 (87.6)	.77	4783 (5.7)	678 (4.6)	<.001
Priority 2	7820 (8.2)	1405 (8.4)		1864 (23.8)	368 (26.2)	.06
Priority 3	2784 (2.9)	499 (3.0)		1218 (43.8)	216 (43.3)	.89
Priority 4	950 (1.0)	170 (1.0)		652 (68.6)	103 (60.6)	.05

^a^ Sequential Organ Failure Assessment score was 0 to 5 for priority 1, 6 to 8 for priority 2, 9 to 11 for priority 3, and ≥12 for priority 4.

^b^ In addition to Sequential Organ Failure Assessment score, systems A and C evaluated chronic comorbidities and patients who were deprioritized and had a heavy burden of chronic comorbidities, which this table does not account for.

^c^ Sequential Organ Failure Assessment score was 0 to 7 for priority 1, 8 to 11 for priority 2, and ≥12 for priority 3.

^d^ Sequential Organ Failure Assessment score was 0 to 8 for priority 1, 9 to 11 for priority 2, 12 to 14 for priority 3, and ≥15 for priority 4.

### Statistical Analysis

The primary outcome was in-hospital mortality. Using the clogit command in Stata (version 16.1; StataCorp), we fit a hierarchical logistic regression model with hospital fixed effects to estimate the within-hospital association of SOFA score as a continuous variable by race with in-hospital mortality.^26^ This model structure controls for unobserved hospital-level confounders in care delivery, given that implementation of CSC would likely occur at the local hospital or regional level. The formula for this model is provided in eAppendix 2 in the Supplement. To determine the association of race and SOFA tiers with mortality, we repeated this analysis with CSC-recommended tiers coded as categorical variables (Table 1). To test the hypothesis that specific SOFA components are associated with overestimation of illness among Black patients, we fit a hierarchical logistic regression model with hospital fixed effects with each SOFA component (ie, respiratory, cardiac, kidney, liver, hematologic, or neurologic components) interacting with race as independent variables associated with in-hospital mortality. We did not adjust for potential confounders (eg, comorbidities) of the association of race and SOFA with mortality in our primary analysis because CSC would not adjust SOFA score in practice. However, we did an exploratory analysis in which we sought to identify key confounders by repeating our hierarchical model with adjustments for age, Charlson Comorbidity Index (CCI) score, and primary admission diagnosis.

Characteristics of White and Black patients were compared using *t* tests, Wilcoxon rank sum tests, and χ^2^ tests, as appropriate. All statistical testing was 2 sided with *P* value thresholds of <.05. All analyses were performed in RStudio version 1.2.5042 (RStudio), R statistical software version 4.0.0 (R Project for Statistical Computing), and Stata. Data were analyzed from May 2020 through April 2021.

#### Sensitivity Analyses

To evaluate the relevance of our findings to a respiratory pandemic, we performed analyses among patients with primary respiratory indications for ICU admission, patients treated in medical ICUs, and patients treated in medical-surgical ICUs. To evaluate potential confounding associated with goals of care, we performed an analysis among patients receiving full therapy only (ie, no do not resuscitate order or other limits on care). To evaluate for confounding by patients who were hospitalized more than once, we performed an analysis with a patient-level variable indicating rehospitalizations and an analysis in which 1 randomly selected hospitalization per patient was included. We performed sensitivity analyses using alternate methods of calculating SOFA (eTable 1 in the Supplement). First, we included only records with Pao_2_ to fraction of inspired oxygen (Fio_2_) ratios, and excluded records calculated with Sao_2_ to Fio_2_ ratio. Second, we calculated cardiac SOFA score with number of vasoactive medications irrespective of dose, given that doses were sometimes reported erroneously. We performed a sensitivity analysis with complete cases only. Dialysis may confound kidney SOFA, and we therefore performed an analysis excluding patients who received dialysis during or prior to SOFA calculation. Additionally, 2 CSC (ie, systems A and C) attempt to prioritize patients with higher long-term as well as short-term survival by deprioritizing patients with both high SOFAs and moderate or severe comorbidities using indices like the CCI.^27^ We performed a sensitivity analysis in which we reclassified patients into priority tiers using SOFA score and comorbidities (eTable 2 in the Supplement).

#### Quantification of Patients at Risk of Inappropriate Deprioritization

We quantified the number of individuals who may be inappropriately deprioritized for resource allocation during CSC implementation, which was associated with overestimation of their mortality risk by SOFA. During CSC implementation, resources are allocated to patients in prioritized tiers.^2,3^ In a severe shortage, sufficient ICU resources would be available for only patients in priority tier 1. In a low shortage situation, conversely, only patients in the bottom tier would be excluded (eFigure 1 in the Supplement). To quantify the number of Black individuals deprioritized by SOFA in SOFA-based CSC, we increased the SOFA threshold required for inclusion in a priority tier for Black patients until hospital-adjusted mortality was equivalent for Black and White patients eligible for resource allocation. We then calculated the number of Black individuals reclassified by this adjustment of SOFA-based tier thresholds.

## Results

### Population Demographic Characteristics and SOFA Score Distribution

**Figure 1.**
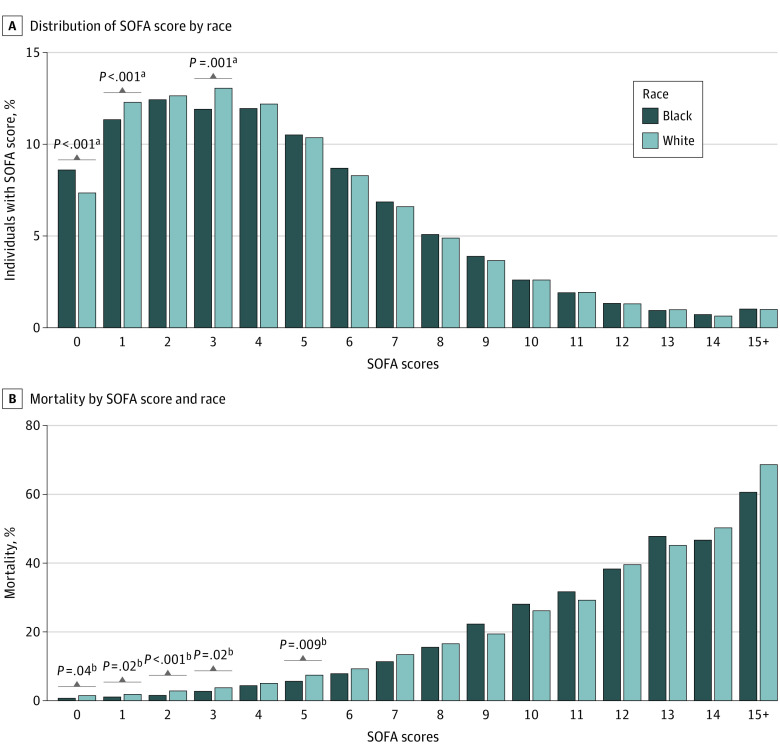
Sequential Organ Failure Assessment (SOFA) Score Distribution and SOFA-Associated Mortality Patients with SOFA scores of 15 or higher were combined, given that there were few patients in this range and this was above the threshold for lowest priority in all Crisis Standards of Care. A, The percentage of Black and White individuals with each SOFA score were calculated. B, The mortality of Black and White individuals with each SOFA score was calculated. ^a^A statistically significantly different proportion of Black and White individuals had a given SOFA score, determined by χ^2^. ^b^A statistically significantly different mortality was found between Black and White individuals at a given SOFA score determined by χ^2^.

Among 111 885 patient encounters representing 95 549 patients, there were 51 464 encounters with women (46.0%) and the mean (SD) age was 63.3 (16.9) years. There were 16 688 encounters with Black patients (14.9%) and 95 197 encounters with White patients (85.1%) in 233 ICUs at 118 hospitals distributed throughout the US (eFigure 2 in the Supplement). Black patients were younger than White patients (mean [SD] age, 56.8 [17.0] years vs 64.5 [16.6] years; *P* < .001), had more comorbidities (mean [SD] CCI score, 0.91 [1.24] vs 0.71 [1.12]; *P* < .001), and were more likely to be treated with full therapy (15 746 patient encounters [94.4%] vs 85 952 patient encounters [90.3%]; *P* < .001) (Table 2). There was no statistically significant difference in the distribution of SOFA scores for White vs Black patients (median [interquartile range], 4 [2-6] for both White and Black individuals; *P* = .19) (Table 2, Figure 1A). Characteristics of the hospitals where encounters for Black and White patients occurred are described in eTable 3 in the Supplement.

**Table 2.** Characteristics of Patient Encounters^a^

Characteristic	Total patient encounters (N = 111 885)	With White patients (n = 95 197 [85.1%])	With Black patients (n = 16 688 [14.9%])	*P* value
SOFA score, median (IQR)				
Total	4 (2-6)	4 (2-6)	4 (2-6)	.19
Respiratory	1 (0-2)	1 (0-2)	0 (0-2)	<.001
Liver	0 (0-0)	0 (0-0)	0 (0-0)	.84
Cardiac	1 (0-1)	1 (0-1)	0 (0-1)	<.001
Kidney	0 (0-2)	0 (0-2)	1 (0-2)	<.001
Neurologic	0 (0-1)	0 (0-1)	0 (0-1)	.01
Hematologic	0 (0-1)	0 (0-1)	0 (0-1)	<.001
Age, mean (SD)	63.3 (16.9)	64.5 (16.6)	56.8 (17.0)	<.001
Women, No. (%)	51 464 (46.0)	43 241 (45.4)	8223 (49.3)	<.001
Medical history, No. (%)				
No chronic health conditions	7882 (7.0)	6801 (7.1)	1081 (6.5)	.002
Neurologic disease	19 868 (17.8)	16 398 (17.2)	3470 (20.8)	<.001
Cardiovascular disease	71 466 (63.9)	60 173 (63.2)	11 293 (67.7)	<.001
Pulmonary disease	25 033 (22.4)	21 336 (22.4)	3697 (22.2)	.40
Gastrointestinal disease	5634 (5.0)	4983 (5.2)	651 (3.9)	<.001
Infectious disease	1700 (1.5)	1212 (1.3)	488 (2.9)	<.001
Hematologic or oncologic disease	17 443 (15.6)	15 366 (16.1)	2077 (12.4)	<.001
Endocrine disease	38 802 (34.7)	32 356 (34.0)	6446 (38.6)	<.001
Rheumatologic disease	2408 (2.2)	2058 (2.2)	350 (2.1)	.59
Kidney disease	14 734 (13.2)	11 327 (11.9)	3407 (20.4)	<.001
Not listed	2879 (2.6)	2475 (2.6)	404 (2.4)	.19
CCI score, mean (SD)	0.74 (1.14)	0.71 (1.12)	0.91 (1.24)	<.001
Primary acute diagnosis by system, No. (%)				
Cardiovascular	49 572 (44.3)	42 494 (44.6)	7078 (42.4)	<.001
Gastrointestinal	10 643 (9.5)	9276 (9.7)	1367 (8.2)	
Genitourinary	2834 (2.5)	2261 (2.4)	573 (3.4)	
Hematologic	737 (0.7)	559 (0.6)	178 (1.1)	
Metabolic or endocrine	4950 (4.4)	3783 (4.0)	1167 (7.0)	
Musculoskeletal or skin	1505 (1.3)	1305 (1.4)	200 (1.2)	
Neurologic	19 313 (17.3)	16 582 (17.4)	2731 (16.4)	
Respiratory	15 178 (13.6)	12 633 (13.3)	2545 (15.3)	
Transplant	198 (0.2)	173 (0.2)	25 (0.1)	
Trauma	4826 (4.3)	4244 (4.5)	582 (3.5)	
Not listed	2129 (1.9)	1887 (2.0)	242 (1.5)	
Full therapy, No. (%)	101 698 (90.9)	85 952 (90.3)	15 746 (94.4)	<.001
Mechanical ventilatory support, No. (%)	39 930 (35.7)	33 958 (35.7)	5972 (35.8)	.78
Survival, No. (%)	102 003 (91.2)	86 680 (91.1)	15 323 (91.8)	.001

Abbreviations: CCI, Charlson Comorbidity Index; IQR, interquartile range; SOFA, Sequential Organ Failure Assessment.

^a^ For hospital characteristics (ie, unit type, size, teaching status, and region) of encounters for Black and White patients, see eTable 3 in the Supplement.

### SOFA Score and Mortality by Race

Black patients had higher in-hospital survival than White patients (15 323 encounters [91.8%] vs 86 680 encounters [91.1%]; *P* = .001). In unadjusted analyses, Black patients had higher survival than White patients when the SOFA score was 0 to 3 or 5 (Figure 1B). In an adjusted analysis with SOFA score as a continuous variable, there was a significant interaction between race and SOFA score, with 2% lower odds of death for Black compared with White patients with equivalent SOFA scores (odds ratio [OR], 0.98; 95% CI, 0.97-0.99; *P* < .001). This finding of a significant interaction between SOFA score and race persisted for all sensitivity analyses, including those that included a patient-level variable indicating multiple hospitalizations (OR, 0.99; 95% CI, 0.98-0.99; *P* < .001) and those that included only 1 randomly selected hospitalization per patient (OR, 0.99; 95% CI, 0.98-0.99, *P* < .001) (eTable 4 in the Supplement). The interaction between race and SOFA remained significant when controlling for CCI score, age, and acute admission diagnosis (OR, 0.99; 95% CI, 0.98-0.996; *P* = .004) (eTable 4 in the Supplement). These analyses were performed using fixed center-level effects instead of random effects because a random effects model was an inconsistent estimator associated with true population parameters according to the Hausman specification test.

### SOFA Components and Mortality Risk

Mortality was lower for Black compared with White individuals with equivalent kidney SOFA scores (OR, 0.91; 95% CI, 0.88-0.95; *P* < .001) but higher at equivalent hematologic SOFA scores (OR, 1.09; 95% CI, 1.01-1.16; *P* = .02). There was no significant difference for respiratory, liver, cardiac, or neurologic SOFA scores (eTable 5, eFigure 3, in the Supplement).

### CSC by Race

**Figure 2.**
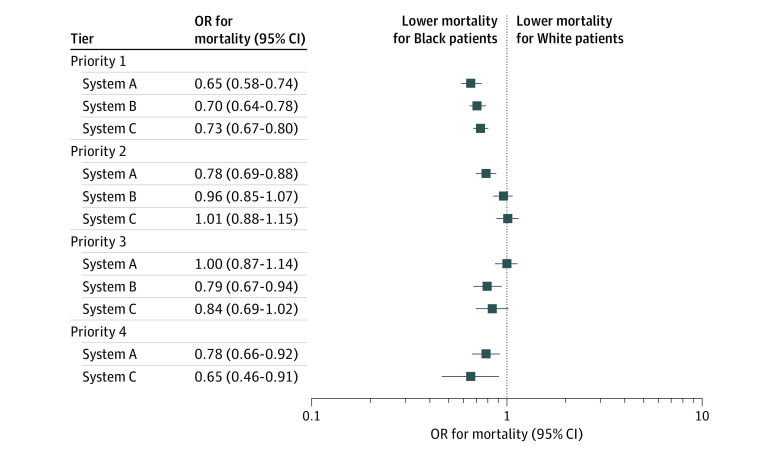
Mortality in Sequential Organ Failure Assessment–Derived Tiers Recommended by Statewide Crisis Standards of Care Hierarchical logistic regression with hospital-level fixed effects was used to calculate the odds ratio (OR) of death for Black compared with White patients with equivalent priority according to crisis standards of care. The odds of death for Black compared with White patients were lower in the highest priority tier (ie, priority 1) and lowest priority tier (ie, priority 3 for system B and priority 4 for systems A and C) of the 3 systems, regardless of the Sequential Organ Failure Assessment score threshold used (ie, for systems A, B, and C, scores of 6, 8, and 9, respectively, for highest priority; 12, 12, and 15, respectively, for lowest priority).

When patients were divided into SOFA tiers, the proportions of White vs Black patient encounters by priority were statistically significantly different in system A (priority 1: 64 774 encounters [68.0%] vs 11 163 encounters [66.9%]; priority 2: 18 869 encounters [19.8%] vs 3451 encounters [20.7%]; priority 3: 7820 encounters [8.2%] vs 1405 encounters [8.4%]; priority 4: 3734 encounters [3.9%] vs 669 encounters [4.0%]; *P* = .03) (Table 1). Compared with White individuals, Black individuals had lower mortality in the highest priority tier of all systems, with a difference of 1.1 percentage points for all systems in unadjusted analyses (system A: 2438 patient encounters with survivals [3.8%] vs 302 patient encounters with survivals [2.7%]; *P* < .001; system B: 4011 patient encounters with survivals [5.1%] vs 546 patient encounters with survivals [4.0%]; *P* < .001; system C: 4783 patient encounters with survivals [5.7%] vs 678 patient encounters with survivals [4.6%]; *P* < .001) (eFigure 4 in the Supplement; Table 1), and in hospital-adjusted analyses (eg, system A: OR, 0.65; 95% CI, 0.58-0.74; *P* < .001; system B: OR, 0.70; 95% CI, 0.64-0.78; *P* < .001; system C: OR, 0.73; 95% CI, 0.67-0.80; *P* < .001) (Figure 2; eTable 6, eTable 7, and eTable 8 in the Supplement). The hospital-adjusted odds of death were lower for Black compared with White patients in the lowest priority tier of all systems (system A: OR, 0.78; 95% CI, 0.66-0.92; *P* = .004; system B: OR, 0.79; 95% CI, 0.67-0.94; *P* = .006; system C: OR, 0.65; 95% CI, 0.46-0.91, *P* = .01) (Figure 2; eTable 6, eTable 7, and eTable 8 in the Supplement). When considering thresholds that would apply in a shortage, Black patients had lower mortality than White patients among patients categorized as high priority at all shortage levels; including severe shortages, intermediate shortages (system A: OR, 0.73; 95% CI, 0.67-0.80; *P* < .001; system B: OR, 0.83; 95% CI, 0.77-0.89; *P* < .001; system C: OR, 0.82; 95% CI, 0.77-0.89; *P* < .001), and low shortages (system A: OR, 0.83; 95% CI, 0.77-0.89; *P* < .001; system C: OR, 0.86; 95% CI, 0.81-0.92; *P* < .001; not applicable for system B, with fewer tiers) (eFigure 1 in the Supplement). In the sensitivity analysis in which priority tiers incorporated comorbidities, statistically significantly more Black than White patients were deprioritized owing to comorbidities (system A: 1596 patient encounters [9.6%] vs 6465 patient encounters [6.8%]; *P* < .001; system C: 33 patient encounters [0.2%] vs 99 patient encounters [0.1%]; *P* = .002), and significant in-hospital mortality differences persisted between White and Black patients in priority tiers consistent with the primary analysis (eTable 9 in the Supplement).

### Quantification of Patients at Risk of Inappropriate Deprioritization

Increasing the SOFA thresholds for Black patients by 2 points equalized the adjusted odds of death for Black and White individuals who qualified for high priority broadly across the 3 systems and under all shortage conditions (eFigure 1 in the Supplement). The lowest rate of inappropriate deprioritization associated with Black patient encounters occurred under low shortage conditions (eg, 379 encounters [2.3%] in system A), although these deprioritizations accounted for 56.7% of 669 patient encounters in which Black patients would be excluded (Table 1, Table 3; eFigure 1 in the Supplement). Substantially more Black patients were misclassified under severe shortage conditions, in which inappropriate deprioritization occurred in up to 2601 encounters for Black patients (15.6%) (Table 3; eFigure 1 and eTable 10 in the Supplement).

**Table 3.** Number of Black Patients Deprioritized by Shortage Condition

System	Patients deprioritized, No. (%)^a^		
	Severe shortage	Intermediate shortage	Low shortage
A	2601 (15.6)	1086 (6.5)	379 (2.3)
B	1501 (9.0)	379 (2.3)	NA^b^
C	1086 (6.5)	379 (2.3)	NA^c^

Abbreviation: NA, not applicable.

^a^ Patients were eligible for allocation if they were priority tier 1 under severe shortage, priority tiers 1 to 2 under intermediate shortage, and priority tiers 1 to 3 under low shortage.

^b^ There were 3 tiers in system B, so only 2 were included during any triage scenario.

^c^ Because of a small number of patients, the total hospital-adjusted Black mortality was equivalent to the hospital-adjusted White mortality at Sequential Organ Failure Assessment score of 14 or less.

## Discussion

This cohort study found that Black individuals had lower actual mortality than White individuals who had equivalent SOFAs, which was associated with a significant structural disadvantage under CSC. This is problematic, given that the ethical rationale for incorporating SOFA scores into CSC is to create an equitable scenario for patients with similar likelihoods of survival. There was a structural disadvantage for Black patients throughout the range of SOFA, suggesting broad relevance to severe and less severe shortages. We controlled for hospital effects in our analysis, effectively considering CSC within a given hospital. Ideally, allocation in a crisis would be regional, with free sharing of resources among hospitals. Unfortunately, the near-activation of CSC in Los Angeles county suggests this would not occur in practice.^28^

Our results corroborate findings from a cohort of patients admitted to the emergency department among whom SOFA was similarly associated with overestimation of mortality for Black compared with White patients, with similar deprioritization of Black patients within CSC.^29^ Our work extends these findings in a separate, regionally diverse population of patients, all of whom required critical care, and suggests that SOFA may persistently overestimate mortality for Black compared with White patients at the onset of critical illness and ICU triage. These findings align with a report that found a higher proportion of members of racial groups other than White who were assigned lowest priority in a CSC triage simulation.^25^ Our results contrast with a report that did not find disparities associated with CSC.^30^ Importantly, that report assessed the proportions of patients in racial groups who were assigned to different priorities without reporting survival differences between racial groups in the priority tiers. Our results highlight the importance of assessing not only proportions, but also short-term survival, a central goal of all CSC.

Our analysis identified significant mortality differences associated with kidney and hematologic SOFA scores among Black and White patients. Differences related to kidney SOFA score may be associated with well-described variation in creatinine as a measure of kidney function in racially diverse populations, or the association may be more complex.^19,20^ Surprisingly, when we adjusted for age, comorbidities, and acute diagnosis, the racial disparity we identified persisted. Race is a social and not a biological category that leads to increased hardship and stress for marginalized groups. Chronic hardship has been associated with dysregulation of the autonomic nervous system, the hypothalamic-pituitary-adrenal axis, and even epigenetic changes that alter immune function.^31,32,33,34,35^ It is plausible that these changes alter the associations between SOFA score and mortality for Black patients, although we cannot confirm this hypothesis with these data.

Our findings may support a need for policy changes. Numerous reports now challenge SOFA as a triage tool. Beyond the problems with racial equity we describe, SOFA has been critiqued for poor discriminant accuracy among patients with pandemic influenza and COVID-19 and for rapid fluctuations that can lead to different triage decisions within hours; furthermore, the clinical significance of mortality differences between SOFA-based tiers has been questioned.^25,36,37,38^ These findings suggest that alternatives or modifications to SOFA should be developed that more equitably predict survival among Black and White patients and should be validated for triage.

Triage systems that fairly allocate resources among individuals may unfairly allocate resources among communities. That is, even if a triage system fairly allocated resources to Black patients in the ICU, it would not account for their likely disproportionate need for ICU resources. One proposal to address this limitation involves preallocating resources while taking structural disadvantage into account. In such reserve systems, valuable resources are reserved for specific categories of individuals (eg, those from disadvantaged zip codes) and triage decisions are deployed within but not between categories.^39,40^ This approach has been successfully used for human organ donation and public school voucher programs.^41^ Another proposal incorporates measures of social disadvantage into allocation guidelines.^42^ For instance, the US National Academies of Science, Engineering, and Medicine incorporated the Social Vulnerability Index (SVI) into its vaccine allocation guideline.^43^ This index ranks census tracts on 15 social factors, such as unemployment rate, minority population, and disability rate, and is used in the guideline as a tiebreaker between equally prioritized individuals.

### Limitations

This study has several limitations. First, we were not able to determine the causal driver of mortality differences we observed. This was not a longitudinal study, and causal inferences should not be drawn. However, for implementation in triage, it is more important to ensure equity than to understand causality. Second, our cohort was not infected with COVID-19, the proximate trigger for recent CSC revisions. Early reports suggest that Black and White patients hospitalized with COVID-19 have equivalent survival.^44,45,46^ While we do not yet know if this association between SOFA score and race applies in the context of COVID-19, it would be reasonable to test these assumptions as data sets become available. Importantly, CSC do not apply to only 1 crisis but to all potential crises that may overwhelm health care resources, including mass casualties and natural disasters. Third, our results are dependent on the joint distribution of SOFA score and race in the eICU sample, which may not be externally generalizable to all populations.

## Conclusions

This study found that mortality associated with SOFA varied by race in a large, multicenter, geographically diverse population of patients admitted to the ICU. These findings may have policy implications for the revision of CSC and for the use of SOFA scores to describe expected mortality when comparing patients in racially diverse populations. Further research should devise alternatives or modifications to SOFA that can be applied within triage algorithms without exacerbating racial disparities.

## Notes

Supplement. **eAppendix 1.** Definitions **eAppendix 2.** Analysis **eTable 1.** Sequential Organ Failure Assessment Score Variations Used in Primary and Sensitivity Analyses by Race **eTable 2.** Triage Priority Levels Incorporating Sequential Organ Failure Assessment and Comorbidities in Triage Systems A and C **eFigure 1.** Reclassification of Black Patients Into Higher Priority After Liberalizing Sequential Organ Failure Assessment Tier Thresholds **eFigure 2.** Derivation of the Cohort **eTable 3.** Additional Patient Characteristics (Supplement to Table 2) **eTable 4.** Hierarchical Conditional Logistic Regression With Fixed Effects by Hospital for Interaction Between Race and Sequential Organ Failure Assessment Score **eTable 5.** Interaction Between Sequential Organ Failure Assessment Components and Race as Predictors Associated With Mortality **eFigure 3.** Association of Sequential Organ Failure Assessment Components With Mortality by Race **eFigure 4.** Mortality by Race in Sequential Organ Failure Assessment–Derived Tiers Recommended by Crisis Standards of Care **eTable 6.** Odds Ratio for Death of Black vs White Individuals With Equivalent Priority, System A **eTable 7.** Odds Ratio for Death of Black vs White Individuals With Equivalent Priority, System B **eTable 8.** Odds Ratio for Death of Black vs White Individuals With Equivalent Priority, System C **eTable 9.** Odds Ratios for Death of Black Compared With White Patients in Priority Tiers That Incorporate Moderate and Severe Comorbidities **eTable 10.** Black Patients Reclassified as Higher Priority by Liberalizing Sequential Organ Failure Assessment Thresholds eReferences
