# Supplementary material for: Accuracy of the Sequential Organ Failure Assessment Score for In-Hospital Mortality by Race and Relevance to Crisis Standards of Care
Source: JAMA Netw Open. 2021 Jun 18;4(6):e2113891. doi: 10.1001/jamanetworkopen.2021.13891 (PMC8214156; doi:10.1001/jamanetworkopen.2021.13891)
Supplement: Supplement. — eAppendix 1. Definitions eAppendix 2. Analysis eTable 1. Sequential Organ Failure Assessment Score Variations Used in Primary and Sensitivity Analyses by Race eTable 2. Triage Priority Levels Incorporating Sequential Organ Failure Assessment and Comorbidities in Triage Systems A and C eFigure 1. Reclassification of Black Patients Into Higher Priority After Liberalizing Sequential Organ Failure Assessment Tier Thresholds eFigure 2. Derivation of the Cohort eTable 3. Additional Patient Characteristics (Supplement to Table 2) eTable 4. Hierarchical Conditional Logistic Regression With Fixed Effects by Hospital for Interaction Between Race and Sequential Organ Failure Assessment Score eTable 5. Interaction Between Sequential Organ Failure Assessment Components and Race as Predictors Associated With Mortality eFigure 3. Association of Sequential Organ Failure Assessment Components With Mortality by Race eFigure 4. Mortality by Race in Sequential Organ Failure Assessment–Derived Tiers Recommended by Crisis Standards of Care eTable 6. Odds Ratio for Death of Black vs White Individuals With Equivalent Priority, System A eTable 7. Odds Ratio for Death of Black vs White Individuals With Equivalent Priority, System B eTable 8. Odds Ratio for Death of Black vs White Individuals With Equivalent Priority, System C eTable 9. Odds Ratios for Death of Black Compared With White Patients in Priority Tiers That Incorporate Moderate and Severe Comorbidities eTable 10. Black Patients Reclassified as Higher Priority by Liberalizing Sequential Organ Failure Assessment Thresholds eReferences [file jamanetwopen-e2113891-s001.pdf]

Supplemental Online Content

Miller WD, Han X, Peek ME, Charan Ashana D, Parker WF. Accuracy of the Sequential Organ Failure Assessment score for in-hospital mortality by race and relevance to crisis standards of care. *JAMA Netw Open*. 2021;4(6):e2113891. doi:10.1001/jamanetworkopen.2021.13891

eAppendix 1. Definitions
eAppendix 2. Analysis
eTable 1. Sequential Organ Failure Assessment Score Variations Used in Primary and Sensitivity Analyses by Race
eTable 2. Triage Priority Levels Incorporating Sequential Organ Failure Assessment and Comorbidities in Triage Systems A and C
eFigure 1. Reclassification of Black Patients Into Higher Priority After Liberalizing Sequential Organ Failure Assessment Tier Thresholds
eFigure 2. Derivation of the Cohort
eTable 3. Additional Patient Characteristics (Supplement to Table 2)
eTable 4. Hierarchical Conditional Logistic Regression With Fixed Effects by Hospital for Interaction Between Race and Sequential Organ Failure Assessment Score
eTable 5. Interaction Between Sequential Organ Failure Assessment Components and Race as Predictors Associated With Mortality
eFigure 3. Association of Sequential Organ Failure Assessment Components With Mortality by Race
eFigure 4. Mortality by Race in Sequential Organ Failure Assessment–Derived Tiers Recommended by Crisis Standards of Care
eTable 6. Odds Ratio for Death of Black vs White Individuals With Equivalent Priority, System A
eTable 7. Odds Ratio for Death of Black vs White Individuals With Equivalent Priority, System B
eTable 8. Odds Ratio for Death of Black vs White Individuals With Equivalent Priority, System C
eTable 9. Odds Ratios for Death of Black Compared With White Patients in Priority Tiers That Incorporate Moderate and Severe Comorbidities
eTable 10. Black Patients Reclassified as Higher Priority by Liberalizing Sequential Organ Failure Assessment Thresholds
eReferences

This supplemental material has been provided by the authors to give readers additional information about their work.

© 2021 Miller WD et al. *JAMA Network Open.*

eAppendix 1. Definitions

Definition of the Included and Excluded Cohort:

(1) *Method to identify first ICU stay during hospitalization, and Transferred patients:* Eligible patientunitstayid's were identified from the Patients table. Records were excluded if the ICU stay was not the first during a hospitalization. The first ICU stay during a hospitalization was identified using the unitvisitnumber field, and was required to be "1". Patients who were transferred were identified using the hospitaladmitsource and unitadmitsource fields. Patients were excluded if the hospitaladmitsource or unitadmitsource was listed as "Other Hospital", "Other ICU", "ICU", or "ICU to SDU". This method excluded 47,538 patients, reducing the number of eligible ICU stays from 200,859 to 153,321.

(2) *Age:* Age was listed in the Patient table. Patients were excluded if they were less than 18 years old (468 patients) or did not have an age listed (84 patients). Patients with an age over 89 had their age set to 90, and were retained (5701 patients). This leaves 152,769 patients.

(3) *Race*: Race was defined based on the "ethnicity" field in the Patient table, in which ethnicity "African American" (17,132 records) or "Caucasian" (116,976 records) was selected from a picklist. Patients were excluded if race was unlisted (1915 records), Asian (2591 records), Hispanic (5901 records), Native American (1092 records), or Other/Unknown (7154 records). Of note, some patients had race listed as "African American" during one admission, and "Caucasian" during a separate admission. These patients were excluded at a later step. Ethnicity was not available as a separate field.

(4) *Mortality*: Mortality was defined based on the hospitaldischargestatus field in the Patient table. Records were retained if hospitaldischargestatus was listed as "Alive" (138,292 records) or "Expired" (13,116 records) and excluded if mortality data was not listed (1,361 records).

(5) *Within 24hrs of ICU admission*: Times in the eICU dataset tables are normalized to the time of ICU admission, listed as an "offset" of 0 (e.g. observationoffset in the vital signs table or labresultoffset in the labs table). Offsets are listed in minutes. To evaluate the SOFA score at the time of ICU admission, we selected values within 24hrs of ICU admission, defined as up to 24hrs prior to ICU admission (offset = -1440), or 24 hours after ICU admission (offset = 1440). This allowed us to assess values that are checked infrequently or daily (such as certain labs).

Cohort characteristics

(6) *Gender:* Defined based on the gender field in the Patient table.

(7) *Limits on Care:* Limits on Care were identified using the cplgroup "Care Limitation" in the Care Plan General table. If the cplitemvalue was set to "Full Therapy", then no limitations on care were inferred. Many patients had different Care Limitation values entered during the course of their stay. Because limits are often placed on care late in the course of illness, after aggressive ICU measures have been attempted but failed, we selected the first recorded value, and performed a sensitivity analysis on this subgroup of patients.

(8) *Acute indication for admission:* Based on the Organ System name listed in the admitdxname field in the admissiondx table.

(9) *Comorbidities:* Based on the organ system listed in the pastHistoryPath field of the pastHistory table.

(10) *Hospital characteristics:* Based on the hospitalid field in the Patient table, and the numbedscategory, teachingstatus (t = "teaching", f = "nonteaching"), and region fields of the hospital table.

(11) *Mechanical Respiratory support:* Defined if the intubated or vent field in the apacheApsVar table was set to "1", or if an FiO2 (as opposed to "LPM O2") was listed in the respiratoryCharting table, or if the treatment listed with the FiO2 in the treatment table was not listed as "nasal cannula", "face mask", "face tent", "non-rebreather mask", or "trach collar".

(12) *ICU type:* based on the unitType field in the Patient table.

(13) *Charlson Comorbidity Index (CCI):* This was defined with ICD9 codes in the diagnosis table based on the definitions of Quan et al.[1] There were 5,382 missing values.

SOFA Score definitions:

Respiratory SOFA:

The PF ratio was the most commonly missing SOFA variable. To calculate the respiratory SOFA, the PF ratio was preferentially used, and when it was not available, the SF ratio was used, as has been described in multiple previous reports.[2–4] The PF ratio was preferentially obtained from the apacheApsVar table (which contains the lowest PF ratio

© 2021 Miller WD et al. *JAMA Network Open.*

from the first 24 hrs of ICU admission). When this was unavailable, the PF ratio was derived by combining a PaO2 from the labs table with an FiO2 from the respiratoryCharting table. The respiratoryCharting table reported an "FiO2", or an "LPM O2". When LPM O2 was reported, the FiO2 was calculated as 0.21 + 0.04*LPM up to 6 LPM. Between 6-20LPM, the FiO2 was set to 0.45, and flows of 20-100LPM were set to FiO2 of 0.60. Flows recorded above 100LPM were considered to be incorrectly entered and were discarded. When FiO2 was not available in the respiratoryCharting table, it was obtained from the treatments table. FiO2 was recorded in the treatments table as a range: <40%, 40-60%, or >60%. These were set to 0.40, 0.60, and 0.70, respectively. For both the respiratoryCharting table and the treatments table values, we used the highest FiO2 recorded within 24hrs of ICU admission. For the PF ratios compiled from both the respiratoryCharting and treatments tables, progressively less stringent time restrictions were imposed as follows: we initially specified that the PaO2 had to be recorded within 90 minutes of the recorded FiO2 value; when a value was not available within 90 minutes, then a value up to 180 minutes after the FiO2 recording was accepted; when this was unavailable, a value up to 6 hrs was accepted; then a value up to 12hrs later, and finally a value up to 24hrs after the recorded FiO2 was accepted. The lowest PF ratio within 24hrs was retained for final analysis, and there were 45,136 PF ratios associated with eligible ICU stays for analysis.

When data was not available to calculate a PF ratio as described above, an SF ratio was calculated. The SF ratio was based preferentially on the FiO2 recorded in the respiratoryCharting table as described above. When this was not available, then the FiO2 was obtained from the treatments table as described above. We specified that the saturation had to be recorded after the FiO2, and we used the saturation recorded soonest after the highest recorded FiO2 within 24hrs of ICU admission. Saturations recorded more than 6hrs after the highest recorded FiO2 were not eligible for consideration. The SF ratio was then calculated and converted to a respiratory SOFA score according to the scale reported by Grissom et al. in their description of the mSOFA for critical care triage.[4] We performed a sensitivity analysis that was restricted to samples with PF ratios only; SF ratios were excluded from this analysis.

Cardiac SOFA:
MAP was preferentially obtained from the meanBp field of the apacheApsVar table. When not available here, it was obtained from the noninvasivemean field of the vitalAperiodic table. The lowest value from within 24 hours of ICU admission was selected. Vasopressors were identified in the infusionDrug table, via identifying generic and trade names of the medications. Vasoactive medications included Dobutamine, Milrinone, Dopamine, Norepinephrine, Epinephrine, Phenylephrine, and Vasopressin. Angiotensin II was not in use at the time of data extraction for this cohort. Many of the vasoactive doses could not be reconciled with standard doses, even after rigorous analysis and cleaning, possibly due to faulty data entry or alternative reporting of dose. Therefore, we performed a sensitivity analysis in which we looked only at the number of vasoactive medications, without taking dose into account. This is similar to methods described by previous investigators.[5] In this analysis, 2 points were assigned for use of Dobutamine, 3 points were assigned for use of 1 vasopressor (Dopamine, Norepinephrine, Epinephrine, Phenylephrine, or Vasopressin), and 4 points were assigned for use of 2 vasopressors.

Liver SOFA:
Bilirubins were preferentially obtained from the apacheApsVar table (values are from the first 24hrs of ICU stay). When not available in the apacheApsVar table, then the highest bilirubin from within 24 hours of ICU admission was obtained from the labs table. When not available in the labs table, then the highest value from the same hospitalization, but prior to 24hrs within ICU admission was accepted.

Kidney SOFA:
Creatinine and urine output were preferentially obtained from the apacheApsVar table. When values were not available there, the creatinine was obtained from the labs table. When not available in the apacheApsVar table, urine output was obtained from the intakeOuput table, by identifying values labeled "urine" or "Nephrostomy". The values from the 24hrs prior to admission were significantly lower than the values from the 24hrs after admission. This was interpreted to likely reflect better recording in the 24hrs after ICU admission. Therefore, Urine output was only accepted from the first 24hrs after ICU admission.

Hematologic SOFA:
Platelet counts were obtained from the lab table. The lowest platelet count within 24hrs of ICU admission was selected, and the SOFA score was calculated using this value.

Neurologic SOFA:

© 2021 Miller WD et al. *JAMA Network Open.*

Glasgow Coma Scale (GCS) was preferentially obtained from the apacheApsVar table (the lowest score in the first 24hrs of ICU admission). If it was not available in this table, then the lowest value from within 24hrs of ICU admission (with simultaneous Verbal, Eyes, and Motor scores) was obtained from the physicalExam table

Missing Values:
Missing values were imputed as SOFA component 0, a standard practice. The rationale is that values are less likely to be checked and documented if normal (i.e. less likely to record FiO2 unless patient is hypoxemic; less likely to check bilirubin unless suspected patholophysiology; less likely to record urine output unless concern that it is abnormal).
The numbers of missing values were as follows:

Missing values in primary analysis:
Respiratory SOFA: 46,363 missing
Liver SOFA: 37,489 missing
Cardiac SOFA: 1,601 missing
Kidney SOFA: 1861 missing
Hematologic SOFA: 6,447 missing
Neurologic SOFA: 3,164 missing

There were 2353 encounters (2.1% of the total) in which the patients died within 24 hours of the index ICU admission. This included 2043 (86.8%) White patients and 310 (13.2%) Black patients, which are approximately similar proportions to the overall cohort. The SOFA score was significantly higher in this subset of patients (median 9, Q1 = 6, Q3 = 12).

Imputation was handled the same for all patients, including those who died within 24 hours. The total number of imputed values for patients who died within 24 hours are as follows: 532 Respiratory SOFAs, 610 hepatic SOFAs, 143 kidney SOFAs, 119 Cardiac SOFAs, 221 Neurologic SOFAs, and 211 Hematologic SOFAs.

© 2021 Miller WD et al. *JAMA Network Open.*

eAppendix 2. Analysis

To test for the effects of our methods of defining SOFA components, we performed sensitivity analyses with two variations of the SOFA score described in eTable 1.

We tested for an interaction between Race and the SOFA score with hierarchical conditional logistic regression, with fixed effects according to hospital using the clogit command in Stata. The formula for this analysis was:

Mortality ~ SOFA+SOFA*Race (grouped by hospital)

To evaluate priority scores (system A, system B, and system C), we used the following formula with the clogit[6] command:

Formula: Mortality~X + X*Race, group(hospital)

X = Priority scores using the thresholds in A, B, or C.

To control for patients who may have been hospitalized multiple times (but who can only die once), we performed one sensitivity analysis with the formula:

Mortality ~ SOFA+SOFA*Race+Multiple_hospitalizations_indicator, group(hospital)

The formula to evaluate the relationship between SOFA components and mortality was:

Mortality~Resp_SOFA+Resp_Race_Interaction + Cardiac_SOFA + Cardiac_Race_Interaction + Kidney_SOFA + Kidney_Race_Interaction + Liver_SOFA + Liver_Race_Interaction + Heme_SOFA + Heme_SOFA_Interaction + Neuro_SOFA + Neuro_SOFA_Interaction, group (hospital)

© 2021 Miller WD et al. *JAMA Network Open.*

eTable 1. Sequential Organ Failure Assessment Score Variations Used in Primary and Sensitivity Analyses by Race

SOFA Score	SOFA Components	White Patients N	Black Patients N	White Median [Q1, Q3]	Black Median [Q1, Q3]	P difference in SOFA
Primary Analysis	Missing values imputed as 0	95,197	16,688	4 [2, 6]	4 [2, 6]	0.19
Indicator variable for multiple hospitalizations	Missing values imputed as 0	95,197	16,688	4 [2, 6]	4 [2, 6]	0.19
Only 1 Hospitalization per patient	Missing values imputed as 0	80,942	13,545	4 [2, 6]	4 [2, 6]	0.76
Restrict to patients who did not receive dialysis	Missing values imputed as 0	92,989	15,506	4 [2, 6]	4 [2, 6]	<0.001
Restrict to MICU	Missing values imputed as 0	8,572	2,002	4 [2, 7]	5 [2, 7]	<0.001
Restrict to Med-Surg	Missing values imputed as 0	47,830	7,365	4 [2, 7]	4 [2, 6]	<0.001
Restrict to Primary Acute Diagnosis is Respiratory	Missing values imputed as 0	12,633	2,545	5 [3, 7]	4 {2, 7]	0.006
Restrict to "Full Therapy"	Missing values imputed as 0	85,952	15,746	4 [2, 6]	4 [2, 6]	0.10
PF ratios only, no SF ratios	Missing values imputed as 0; cases with missing PF ratios were excluded	30,415	5,024	6 [4, 9]	6 [4, 9]	0.16
Number of Vasoactives only; doses ignored	Missing values imputed as 0	95,197	16,688	4 [2, 6]	46 [2, 6]	0.11
Complete Cases Only	No missing values	36,760	6,336	6 [6, 8]	6 [4, 8]	0.67

eTable 1 Legend: We tested 3 variations of the SOFA score. The SOFA score tested in the primary analysis is described in the text. We also performed a sensitivity analysis in which the respiratory SOFA was based on PF ratio only, without inclusion of SOFA scores based on the SF ratio. We also tested a variation of the Cardiac SOFA in which the score was based on number of vasoactive medications irrespective of dose. Sensitivity analyses included (1) incorporating a patient-level indicator variable for patients who had multiple hospitalizations, (2) including only 1 randomly selected hospitalization per patient, (3) including only patients who did not receive dialysis during or prior to SOFA calculation, (4) including only patients treated in Medical ICUs, or Medical-Surgical ICUs, (5) including only patients whose primary admission diagnosis was respiratory failure, (6) including only patients treated with "Full Therapy" (no DNR or other limitations), and (7) analyzing complete cases only. Differences in the medians were tested with the Wilcoxon rank-sum test. *Abbreviations:* N, number; Q1, 25th percentile; Q3, 75th percentile

© 2021 Miller WD et al. *JAMA Network Open.*

eTable 2. Triage Priority Levels Incorporating Sequential Organ Failure Assessment and Comorbidities in Triage Systems A and C

**System A***			
1 Point	2 Points	3 Points	4 Points
SOFA <6	SOFA 6-8	SOFA 9-11	SOFA>11
	Moderate Comorbidity (CCI ≥ 3)^		Major Comorbidity (CCI ≥ 8)^^
**System C****			
1 Point	2 Points	3 Points	4 Points
SOFA <9	SOFA 9-11	SOFA 12-14	SOFA>14
		Major Comorbidity (CCI ≥ 8)^^^	

***** System A is reorganized into 3 priority tiers: High priority (1-3 total SOFA tier +Comorbidity points), Intermediate priority (4-5 SOFA tier + Comorbidity points), and Low priority (5-8 SOFA tier + Comorbidity points)

** System C does not recommend reorganization into tiers after adding SOFA and Comorbidity points. For analysis, we used 1 point, 2 points, 3 points, and 4+ points (points coming from SOFA tiers or Comorbidity points), due to the very small number of patients who were reclassified based on comorbidities.

^1,563 Black patient encounters & 6,366 White patient encounters received 2 points for CCI 3.

^^33 Black patient encounters & 99 White patient encounters received 4 points for CCI 8.

^^^33 Black patient encounters & 99 White patient encounters received 3 points for CCI 8.

© 2021 Miller WD et al. *JAMA Network Open.*

eFigure 1. Reclassification of Black Patients Into Higher Priority After Liberalizing Sequential Organ Failure Assessment Tier Thresholds

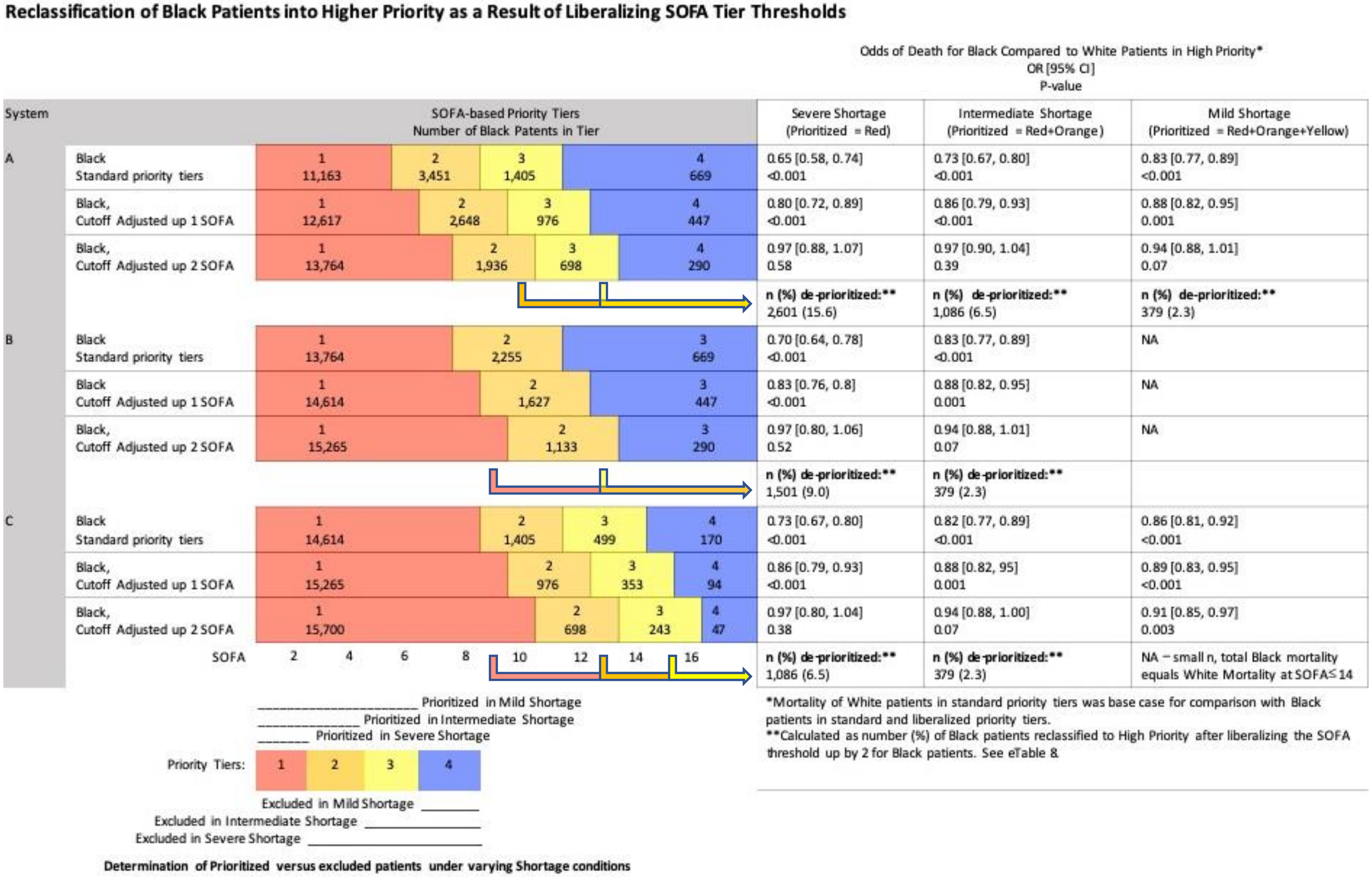

eFigure 1 Legend: During CSC implementation, resources are allocated only to patients in higher priority tiers, and patients in other tiers are excluded. The number of included tiers is determined by available resources (e.g. in a severe shortage, there are only enough resources for Priority 1 patients, but in an intermediate shortage there may be enough resources for patients in Priorities 1 & 2, etc.). To determine the number of Black patients deprioritized in SOFA tiers, we raised the SOFA threshold for inclusion in a priority tier until hospital-adjusted mortality was equal for Black and White patients who were eligible for resource allocation. We then calculated the number of Black patients reclassified by this process.

© 2021 Miller WD et al. *JAMA Network Open.*

eFigure 2. Derivation of the Cohort

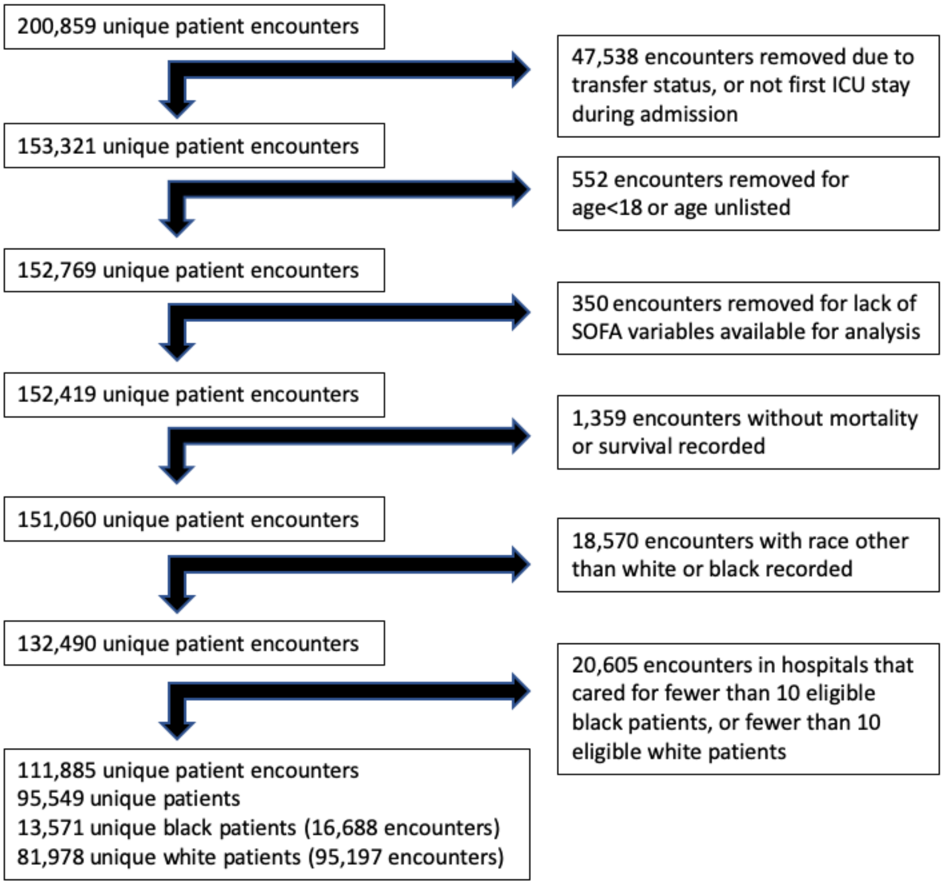

© 2021 Miller WD et al. *JAMA Network Open.*

eTable 3. Additional Patient Characteristics (Supplement to Table 2)

	Total<br>n= 111,885<br>(100%)	Black<br>n = 16,688<br>(14.9%)	White<br>n = 95,197<br>(85.1%)	p-value
Unit type, n (%)				<0.001
- Cardiac ICU	8,177 (7.3)	1,974 (11.8)	6,203 (6.5)	
- CCU-CTICU	11,193 (10.0)	1,371 (8.2)	9,822 (10.3)	
- C-SICU	4,889 (4.4)	236 (1.4)	4,653 (4.9)	
- CT-ICU	4,105 (3.7)	875 (5.2)	3,230 (3.4)	
- Med-Surg ICU	55,195 (49.3)	7,365 (44.1)	47,830 (50.2)	
- MICU	10,574 (9.5)	2,002 (12.0)	8,572 (9.0)	
- Neuro ICU	9,654 (8.6)	1,403 (8.4)	8,251 (8.7)	
- SICU	8,098 (7.2)	1,462 (8.8)	6,636 (7.0)	
Hospital size (beds), n (%)				<0.001
- Less than 100	3,035 (2.7)	292 (1.7)	2,743 (2.9)	
- 100 to 249	20,459 (18.3)	3,299 (19.8)	17,160 (18.0)	
- 250 to 499	25,608 (22.9)	3,221 (19.3)	22,387 (23.5)	
- More than 500	48,865 (43.7)	7,868 (47.1)	40,997 (43.1)	
- Unlisted	13,918 (12.4)	2,008 (12.0)	11,910 (12.5)	
Teaching Hospital, n (%)	32,984 (29.5)	5,509 (33.0)	27,475 (28.9)	<0.001
Hospital Region				<0.001
- Midwest	39,117 (35.0)	4,564 (27.3)	34,553 (36.3)	
- Northeast	8,236 (7.4)	300 (1.8)	7,936 (8.3)	
- South	41,223 (36.8)	9,830 (58.9)	31,393 (33.0)	
- West	18,131 (16.2)	1,371 (8.2)	16,760 (17.6)	
- Unlisted	5,178 (4.6)	623 (3.7)	4,555 (4.8)	

Abbreviations: CCU-CTICU, Cardiac and Cardiothoracic Surgery ICU; C-SICU, Cardiac Surgery ICU; CT-ICU, Cardiothoracic ICU; Med-Surg ICU, Medical-Surgical combined ICU; MICU, Medical ICU; SICU, Surgical ICU.

© 2021 Miller WD et al. *JAMA Network Open.*

eTable 4. Hierarchical Conditional Logistic Regression With Fixed Effects by Hospital for Interaction Between Race and Sequential Organ Failure Assessment Score

Model	Odds Ratio SOFA [95% CI]	p	Odds Ratio Interaction [95%CI]	p
Primary analysis^	1.39 [1.39, 1.40]	<0.001	0.98 [0.97, 0.99]	<0.001
Indicator variable for multiple hospitalizations^	1.40 [1.39, 1.41]	<0.001	0.99 [0.98, 0.99]	<0.001
Restrict to one hospitalization per patient^	1.39 [1.38, 1.40]	<0.001	0.99 [0.98, 0.99]	0.002
Restrict to patients who did not receive dialysis	1.40 [1.39, 1.41]	<0.001	0.98 [0.98, 0.99]	0.001
Restrict to MICU	1.39 [1.36, 1.42]	<0.001	0.96 [0.94, 0.98]	0.001
Restrict to Med-Surg	1.39 [1.38, 1.40]	<0.001	0.97 [0.96, 0.99]	<0.001
Restrict to Primary Acute Diagnosis is Respiratory	1.32 [1.30, 1.34]	<0.001	0.94 [0.92, 0.96]	<0.001
Restrict to “Full Therapy”	1.41 [1.40, 1.42]	<0.001	0.98 [0.98, 0.99]	0.001
Assess Pressor number but not dose (modified Cardiac SOFA)	1.40 [1.39, 1.41]	<0.001	0.98 [0.97, 0.99]	<0.001
Assess PF ratio only, not SF ratio	1.33 [1.32, 1.35]	<0.001	0.99 [0.98, 0.99]	0.02
Complete Cases Only	1.37 (1.36, 1.39]	<0.001	0.98 [0.97, 0.99]	<0.001
Adjust for Charlson Comobidity Index (CCI)*	1.40 [1.39, 1.41]	<0.001	0.97 [0.96, 0.98]	<0.001
Adjust for CCI & age**	1.40 [1.39, 1.41]	<0.001	0.99 [0.98, 0.996]	0.005
Adjust for CCI, age, admit diagnosis ***	1.40 [1.39, 1.41]	<0.001	0.99 [0.98, 0.996]	0.004

*Formula for this model: Mortality~SOFA+SOFA*Race+CCI, (stratified by hospital)
**Formula for this model: Mortality~SOFA+SOFA*Race+age+CCI (stratified by hospital)
***Formula for this model: Mortality~SOFA+SOFA*Race+age+CCI+admit-dx (stratified by hospital)
Legend: In these models, race was coded as 1 for Black individuals and 0 for White individuals, such that a coefficient of interaction of less than 1 indicates lower mortality for Black compared to White individuals.
Abbreviations: 95% CI, 95% Confidence Interval

Coefficients [95% CI], p-value for variables besides Race, SOFA, and Race-SOFA Interaction:
CCI: 1.16 [1.14, 1.18], <0.001
Age: 1.03 [1.03, 1.03], <0.001
Admit diagnoses:

- Cardiovascular (base)
- Gastrointestinal: 0.78 [0.71, 0.85], <0.001
- Genitourinary: 0.47 [0.40, 0.57], <0.001
- Hematologic: 1.05 [0.79, 1.39], 0.74
- Metabolic/Endocrine: 0.42 [0.33, 0.52], <0.001
- Musculoskeletal/Skin: 0.62 [0.47, 0.82], 0.001
- Neurologic: 1.09 [1.01, 1.17], 0.03
- Respiratory: 1.17 [1.10, 1.26], <0.001
- Transplant: 0.03 [0.01, 0.11], <0.001
- Trauma: 1.24 [1.09, 1.41], 0.001

^Hausman specification test was performed to compare random-effects with fixed-effects models in these three cases, and suggested that random-effects models were inconsistent estimators of true population parameters.

eTable 5. Interaction Between Sequential Organ Failure Assessment Components and Race as Predictors Associated With Mortality

SOFA Component	Odds Ratio for Mortality with 1-point increase in SOFA Component among White individuals [95% CI]	Odds Ratio for Mortality in Black compared to White individuals with Equal Score in SOFA Component [95% CI]	P (Mortality in Black Compared to White Individuals)
Respiratory	1.35 [1.32, 1.37]	0.97 [0.93, 1.02]	0.22
Liver	1.45 [1.40, 1.50]	1.08 [0.99, 1.17]	0.09
Cardiac	1.34 [1.31, 1.36]	0.99 [0.94, 1.04]	0.63
Kidney	1.35 [1.33, 1.37]	0.91 [0.88, 0.95]	<0.001
Neurologic	1.63 [1.60, 1.66]	0.98 [0.94, 1.02]	0.33
Hematologic	1.19 [1.15, 1.22]	1.09 [1.01, 1.16]	0.02

Legend: Formula tested was Mortality~Respiratory_SOFA + Respiratory_SOFA*Race + Liver_SOFA + Liver_SOFA*Race + Cardiac_SOFA + Cardiac_SOFA*Race + Kidney_SOFA + Kidney_SOFA*Race + Neurologic_SOFA + Neurologic_SOFA*Race + Hematologic_SOFA+Hematologic_SOFA*Race. This formula was tested within a hierarchical logistic regression framework with a fixed effect term for hospital.
Abbreviations: 95% CI, 95% Confidence Interval

© 2021 Miller WD et al. *JAMA Network Open.*

eFigure 3. Association of Sequential Organ Failure Assessment Components with Mortality, by Race

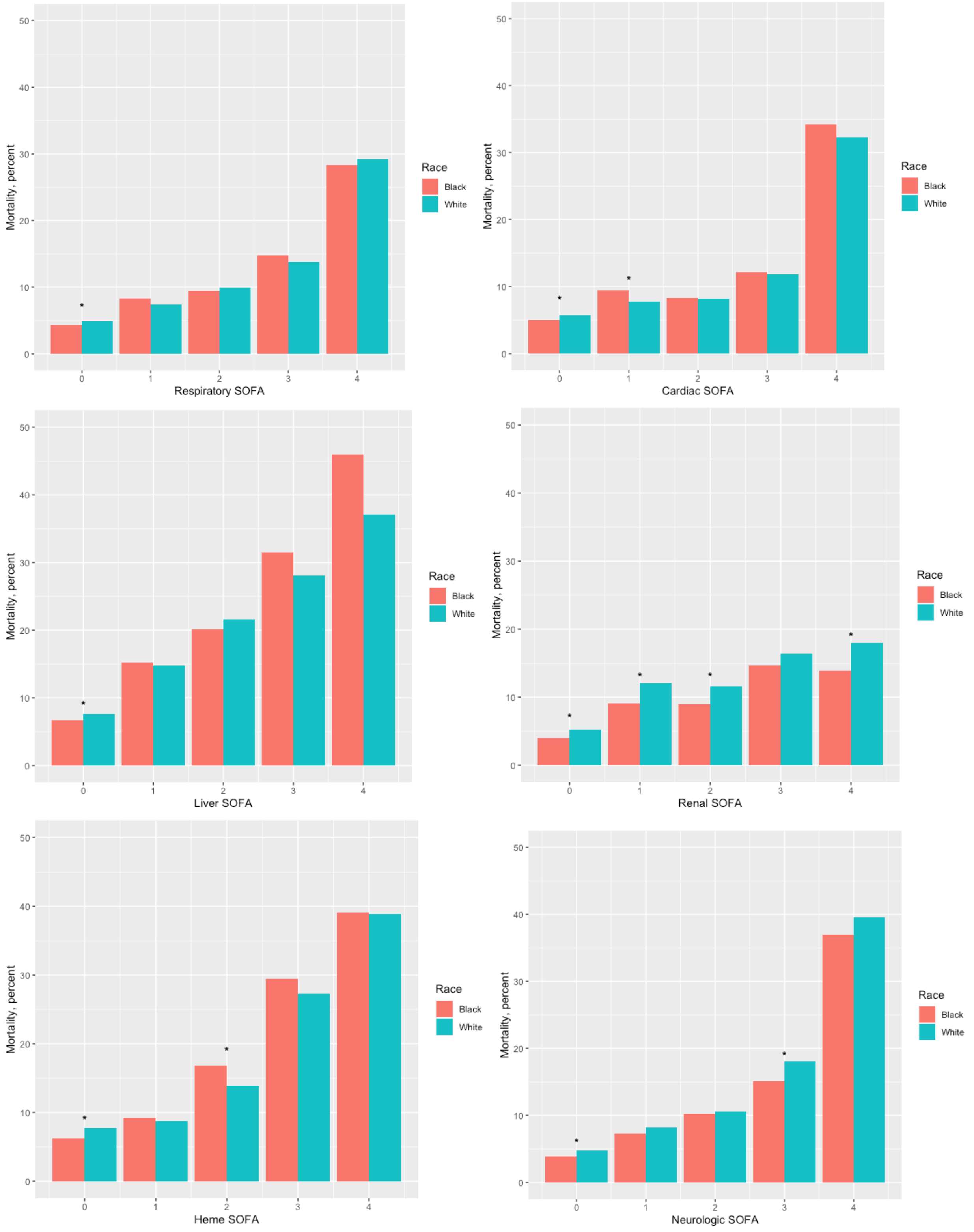

Legend: next page

© 2021 Miller WD et al. *JAMA Network Open.*

Legend eFigure 3:

Mortality was calculated for Black and White patients associated with each SOFA component score: (a) Respiratory SOFA, (b) Cardiac SOFA, (c) Liver SOFA, (d) Kidney (renal) SOFA, (e) Hematologic SOFA, and (f) Neurologic SOFA. * indicates significance ($p<0.05$) by chi-square to test for differences in mortality between Black and White patients with the same SOFA component score. Results of the adjusted analysis are presented in the text and this supplement (eTable 4). In the adjusted analysis, only the Kidney (renal) and Hematologic components were significantly associated with race as predictors of mortality.

© 2021 Miller WD et al. *JAMA Network Open.*

eFigure 4. Mortality by Race in Sequential Organ Failure Assessment–Derived Tiers Recommended by Crisis Standards of Care

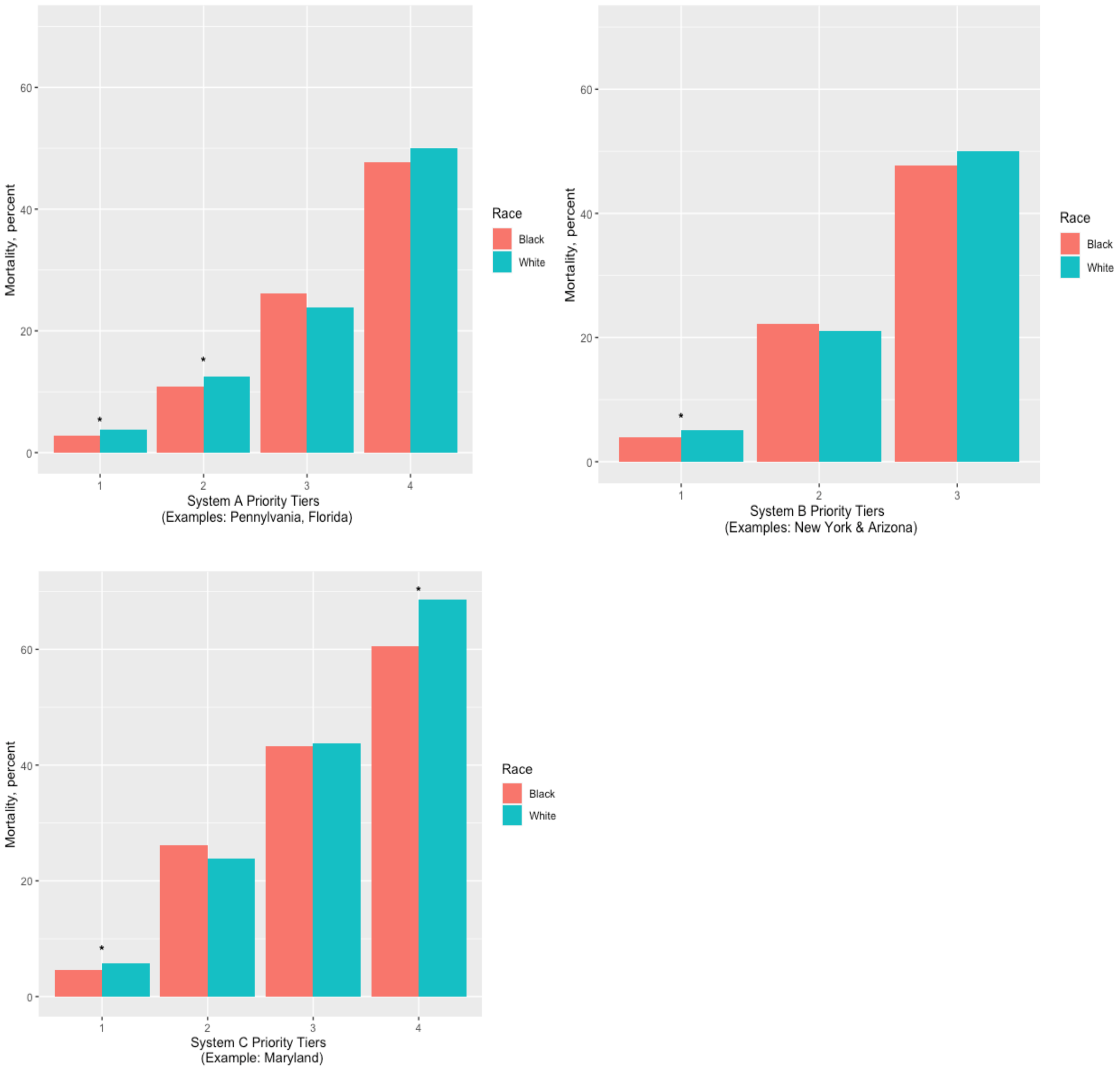

*indicates significance p<0.05 in unadjusted analysis

Legend: (a-c) Mortality for Black and White patients within priority tiers were calculated for 3 CSCs that rely on different SOFA-based thresholds to determine priority for ICU resources during a shortage. * indicates significance (p<0.05) by chi-square to test for differences in mortality between Black and White individuals within individual priority tiers, unadjusted analysis.

© 2021 Miller WD et al. *JAMA Network Open.*

eTable 6. Odds Ratio for Death of Black vs White Individuals With Equivalent Priority, System A

SOFA version	Priority 1<br>Odds Ratio of Death for Black Compared to White Individuals<br>[95% CI]<br>(p)	Priority 2<br>Odds Ratio of Death for Black Compared to White Individuals<br>[95% CI]<br>(p)	Priority 3<br>Odds Ratio of Death for Black Compared to White Individuals<br>[95% CI]<br>(p)	Priority 4<br>Odds Ratio of Death for Black Compared to White Individuals<br>[95% CI]<br>(p)
Primary Analysis	0.65<br>[0.58, 0.74]<br><0.001	0.78<br>[0.69, 0.88]<br><0.001	1.00<br>[0.87, 1.14]<br>0.94	0.78<br>[0.66, 0.92]<br>0.004
Multiple Hospitalizations Indicated	0.68<br>[0.60, 0.77]<br><0.001	0.82<br>[0.73, 0.93]<br>0.001	1.05<br>[0.92, 1.21]<br>0.44	0.82<br>[0.69, 0.97]<br>0.02
One hospitalization per patient	0.64<br>[0.56, 0.73]<br><0.001	0.80<br>[0.70, 0.91]<br>0.001	1.10<br>[0.96, 1.28]<br>0.18	0.83<br>[0.69, 1.00]<br>0.05
Restrict to patients who did not receive dialysis	0.65<br>[0.57, 0.74]<br><0.001	0.81<br>[0.71, 0.91]<br>0.001	1.07<br>[0.93, 1.24]<br>0.32	0.81<br>[0.67, 0.98]<br>0.03
Cardiac SOFA variation (# of vasopressors)	0.65<br>[0.58, 0.74]<br><0.001	0.78<br>[0.69, 0.87]<br><0.001	1.00<br>[0.87, 1.14]<br>0.99	0.75<br>[0.63, 0.90]<br>0.001
Respiratory SOFA variation (PF ratios only)	0.66<br>[0.53, 0.81]<br><0.001	0.87<br>[0.75, 1.02]<br>0.09	1.08<br>[0.92, 1.27]<br>0.33	0.79<br>[0.66, 0.96]<br>0.02
Complete Cases Only	0.54<br>[0.43, 0.68]<br><0.001	0.74<br>[0.63, 0.88]<br><0.001	1.00<br>[0.85, 1.17]<br>0.96	0.77<br>[0.64, 0.93]<br>0.008

Legend eTable 6: Hospital-adjusted Odds of Death for Black Compared to White patients with equal SOFA-related priority was determined for SOFA tiers recommended by System A (examples of states recommending this tier system: Pennsylvania, Florida). Sensitivity analyses were describd in the text, and included (1) a model with a patient-level variable indicating whether a patient had been hospitalized more than once, (2) a model fit by including only one randomly selected hospitalization per patient, (3) excluding any patients who received dialysis during or prior to SOFA calculation, (4) a calculation relying only on number of vasoactive medications irrespective of doses, (5) a SOFA calculation relying only on PF ratios and excluding records that did not have a PF ratio available for calculation, and (6) a SOFA calculation using complete cases only, without any missing values.
Abbreviations: SOFA, Sequential Organ Failure Assessment; PF ratio, PaO2 to FiO2 ratio; CI, confidence interval

© 2021 Miller WD et al. *JAMA Network Open.*

eTable 7. Odds Ratio for Death of Black vs White Individuals With Equivalent Priority, System B

SOFA version	Priority 1 Odds Ratio of Death for Black Compared to White Individuals [95% CI] (p)	Priority 2 Odds Ratio of Death for Black Compared to White Individuals [95% CI] (p)	Priority 3 Odds Ratio of Death for Black Compared to White Individuals [95% CI] (p)
Primary analysis	0.70 [0.64, 0.78] <0.001	0.96 [0.85, 1.07] 0.43	0.79 [0.67, 0.94] 0.006
Multiple Hospitalizations Indicated	0.74 [0.67, 0.81] <0.001	1.01 [0.90, 1.13] 0.84	0.83 [0.69, 0.98] 0.03
One Hospitalization per patient	0.70 [0.63, 0.77] <0.001	1.05 [0.93, 1.19] 0.42	0.84 [0.70, 1.01] 0.07
Restrict to patients who did not receive dialysis	0.70 [0.64, 0.78] <0.001	1.02 [0.91, 1.15] 0.70	0.82 [0.68, 0.99] 0.04
Cardiac SOFA variation (# of vasopressors)	0.71 [0.64, 0.77] <0.001	0.96 [0.85, 1.07] 0.42	0.76 [0.64, 0.91] 0.002
Respiratory SOFA variation (PF ratios only)	0.74 [0.65, 0.86] <0.001	1.06 [0.92, 1.21] 0.42	0.80 [0.66, 0.97] 0.02
Complete Cases Only	0.62 [0.53, 0.72] <0.001	0.96 [0.84, 1.10] 0.57	0.78 [0.64, 0.94] 0.008

Legend eTable 7: Hospital-adjusted Odds of Death for Black Compared to White patients with equal SOFA-related priority was determined for SOFA tiers recommended by System B (examples of states recommending this tier system: New York, Arizona). Sensitivity analyses were describd in the text, and included (1) a model with a patient-level variable indicating whether a patient had been hospitalized more than once, (2) a model fit by including only one randomly selected hospitalization per patient, (3) excluding any patients who received dialysis during or prior to SOFA calculation, (4) a calculation relying only on number of vasoactive medications irrespective of doses, (5) a SOFA calculation relying only on PF ratios and excluding records that did not have a PF ratio available for calculation, and (6) a SOFA calculation using complete cases only, without any missing values.
Abbreviations: SOFA, Sequential Organ Failure Assessment; PF ratio, PaO2 to FiO2 ratio; CI, confidence interval

© 2021 Miller WD et al. *JAMA Network Open.*

eTable 8. Odds Ratio for Death of Black vs White Individuals With Equivalent Priority, System C

SOFA version	Priority 1<br>Odds Ratio of Death for Black Compared to White Individuals<br>[95% CI]<br>(p)	Priority 2<br>Odds Ratio of Death for Black Compared to White Individuals<br>[95% CI]<br>(p)	Priority 3<br>Odds Ratio of Death for Black Compared to White Individuals<br>[95% CI]<br>(p)	Priority 4<br>Odds Ratio of Death for Black Compared to White Individuals<br>[95% CI]<br>(p)
Primary analysis	0.73<br>[0.67, 0.80]<br><0.001	1.01<br>[0.88, 1.15]<br>0.92	0.84<br>[0.69, 1.02]<br>0.08	0.65<br>[0.46, 0.91]<br>0.01
Multiple Hospitalizations Indicated	0.76<br>[0.70, 0.83]<br><0.001	1.06<br>[0.93, 1.22]<br>0.38	0.87<br>[0.72, 1.07]<br>0.19	0.69<br>[0.49, 0.97]<br>0.03
One Hospitalization per patient	0.73<br>[0.67, 0.80]<br><0.001	1.12<br>[0.97, 1.29]<br>0.14	0.90<br>[0.73, 1.12]<br>0.36	0.69<br>[0.47, 1.00]<br>0.05
Restrict to patients who did not receive dialysis	0.73<br>[0.67, 0.80]<br><0.001	1.09<br>[0.95, 1.26]<br>0.24	0.90<br>[0.72, 1.11]<br>0.32	0.62<br>[0.42, 0.90]<br>0.01
Cardiac SOFA variation (# of vasopressors)	0.73<br>[0.67, 0.80]<br><0.001	1.01<br>[0.89, 1.16]<br>0.86	0.77<br>[0.63, 0.94]<br>0.01	0.73<br>[0.51, 1.06]<br>0.10
Respiratory SOFA variation (PF ratios only)	0.79<br>[0.70, 0.90]<br><0.001	1.09<br>[0.93, 1.29]<br>0.27	0.87<br>[0.70, 1.09]<br>0.24	0.61<br>[0.42, 0.88]<br>0.009
Complete Cases Only	0.68<br>[0.60, 0.78]<br><0.001	1.00<br>[0.85, 1.17]<br>0.95	0.84<br>[0.67, 1.04]<br>0.11	0.63<br>[0.44, 0.91]<br>0.01

Abbreviations: PA, Pennsylvania; FL, Florida; NY, New York; AZ, Arizona; MD, Maryland; PF ratio, PaO2/FiO2 ratio; p, p-value

References

Legend eTable 8: Hospital-adjusted Odds of Death for Black Compared to White patients with equal SOFA-related priority was determined for SOFA tiers recommended by System C (examples of states recommending this tier system: Maryland). Sensitivity analyses were describd in the text, and included (1) a model with a patient-level variable indicating whether a patient had been hospitalized more than once, (2) a model fit by including only one randomly selected hospitalization per patient, (3) excluding any patients who received dialysis during or prior to SOFA calculation, (4) a calculation relying only on number of vasoactive medications irrespective of doses, (5) a SOFA calculation relying only on PF ratios and excluding records that did not have a PF ratio available for calculation, and (6) a SOFA calculation using complete cases only, without any missing values.

Abbreviations: SOFA, Sequential Organ Failure Assessment; PF ratio, PaO2 to FiO2 ratio; CI, confidence interval

© 2021 Miller WD et al. *JAMA Network Open.*

eTable 9. Odds Ratios for Death of Black Compared With White Patients in Priority Tiers That Incorporate Moderate and Severe Comorbidities

	Priorities Based on SOFA + Comorbidity Points (see eTable 2)			
	Priority 1	Priority 2	Priority 3	Priority 4
System A	0.80 [0.74, 0.87]<br><0.001	0.71 [0.62, 0.82]<br><0.001	0.65 [0.44, 0.94]<br>0.02	NA
System C	0.73 [0.67, 0.80]<br><0.001	1.01 [0.89, 1.16]<br>0.82	0.85 [0.69, 1.31]<br>0.10	0.57 [0.42, 0.78]<br><0.001

Legend: As described in the main text and eTable 2, Systems A & C attempt to save life-years as well as lives by de-prioritizing patients with life-limiting moderate or severe comorbidities. We performed a sensitivity analysis in which we added comorbidity points and re-classified patients' priorities based on both SOFA and Comorbidity points as recommended in these guidelines. Thresholds for "moderate" and "severe" comorbidities were CCI of 3 and 8, respectively, as recommended in the Crisis Standard of Care guideline from Colorado. Significant differences in in-hospital mortality persisted between Black and White patients in systems A & C after incorporation of comorbidities. Importantly, improving in-hospital mortality is not the goal of incorporating comorbidities.

© 2021 Miller WD et al. *JAMA Network Open.*

eTable 10. Black Patients Reclassified as Higher Priority by Liberalizing Sequential Organ Failure Assessment Thresholds

	N (%) Black Patients Qualifying as High Priority During:		
	**Severe Shortage (Priority 1)**	**Intermediate Shortage (Priorities 1+2)**	**Mild Shortage (Priorities 1-3)**
**System A**	**SOFA 0-5**	**SOFA 0-8**	**SOFA 0-11**
Standard Tier Definition	11, 163 (66.9)	14,614 (87.6)	16,019 (96.0)
Adjust SOFA threshold up by 1	12,617 (75.6)	15,265 (91.5)	16,241 (97.3)
Adjust SOFA threshold up by 2	13,764 (82.5)	15,700 (94.1)	16,398 (98.3)
N(%) Black patients reassignged by SOFA adjustment*	2,601 (15.6)	1,086 (6.5)	379 (2.3)
White Versus Black Mortality in Tier after Priority Threshold Adjustment for Black Individuals	3.8 vs 4.0	5.7 vs 6.0	7.3 vs 7.4
**System B**	**SOFA 0-7**	**SOFA 0-11**	**NA**
Standard Tier Definitions	13,764 (82.5)	16,019 (96.0)	
Adjust SOFA threshold up by 1 if Black	14,614 (87.6)	16,241 (97.3)	
Adjust SOFA threshold up by 2 if Black	15,265 (91.5)	16,398 (98.3)	
N(%) Black patients reassignged by SOFA adjustment*	1,501 (9.0)	379 (2.3)	
White Versus Black Mortality in Tier after Priority Threshold Adjustment for Black Individuals	5.1 vs 5.4	7.3 vs 7.4	
**System C**	**SOFA 0-8**	**SOFA 0-11**	**SOFA 0-14**
Standard Tier Definitions	14,614 (87.6)	16,019 (96.0)	16,518 (99.0)
Adjust SOFA threshold up by 1 if Black	15,265 (91.5)	16,241 (97.3)	16,594 (99.4)
Adjust SOFA threshold up by 2 if Black	15,700 (94.1)	16,398 (98.3)	16,641 (99.7)
N(%) Black patients reassignged by SOFA adjustment*	1,086 (6.5)	379 (2.3)	123 (0.7)
White Versus Black Mortality in Tier after Priority Threshold Adjustment for Black Individuals	5.7 vs 6.0	7.3 vs 7.4	8.3 vs 8.0

*SOFA threshold adjusted up by 2 for Black patients (e.g. eligible for highest priority with SOFA 7 instead of 5 in System A); abbreviations: N, Number; SOFA, Sequential Organ Failure Assessment

© 2021 Miller WD et al. *JAMA Network Open.*

eReferences

1. Quan H, Sundararajan V, Halfon P, et al. Coding Algorithms for Defining Comorbidities in ICD-9-CM and ICD-10 Administrative Data: *Med Care*. 2005;43(11):1130-1139. doi:10.1097/01.mlr.0000182534.19832.83

2. van den Boom W, Hoy M, Sankaran J, et al. The Search for Optimal Oxygen Saturation Targets in Critically Ill Patients. *Chest*. 2020;157(3):566-573. doi:10.1016/j.chest.2019.09.015

3. Pandharipande PP, Shintani AK, Hagerman HE, et al. Derivation and validation of Spo2/Fio2 ratio to impute for Pao2/Fio2 ratio in the respiratory component of the Sequential Organ Failure Assessment score*: *Crit Care Med*. 2009;37(4):1317-1321. doi:10.1097/CCM.0b013e31819cefa9

4. Grissom CK, Brown SM, Kuttler KG, et al. A Modified Sequential Organ Failure Assessment Score for Critical Care Triage. *Disaster Med Public Health Prep*. 2010;4(4):277-284. doi:10.1001/dmp.2010.40

5. Seymour CW, Liu VX, Iwashyna TJ, et al. Assessment of Clinical Criteria for Sepsis: For the Third International Consensus Definitions for Sepsis and Septic Shock (Sepsis-3). *JAMA*. 2016;315(8):762. doi:10.1001/jama.2016.0288

6. McFadden D. Conditional logit analysis of qualitative choice behavior. In: *Frontiers in Econometrics*. Academic Press; 1973:105-142. Accessed April 4, 2021. https://eml.berkeley.edu/reprints/mcfadden/zarembka.pdf

© 2021 Miller WD et al. *JAMA Network Open.*
